# Detection of EGFR Mutations in Cerebrospinal Fluid of EGFR-Mutant Lung Adenocarcinoma With Brain Metastases

**DOI:** 10.3389/fonc.2021.622142

**Published:** 2021-03-22

**Authors:** Liang Shi, Junfang Tang, Hong Tao, Lili Guo, Weihua Wu, Hongbo Wu, Zichen Liu, Li Tong, Wei Wu, Hongxia Li, Qiyi Meng, Liyan Xu, Nanying Che, Zhe Liu

**Affiliations:** ^1^ Department of Medical Oncology, Beijing Chest Hospital, Capital Medical University, Beijing Tuberculosis and Thoracic Tumor Research Institute, Beijing, China; ^2^ Department of Pathology, Beijing Chest Hospital, Capital Medical University, Beijing Tuberculosis and Thoracic Tumor Research Institute, Beijing, China

**Keywords:** lung adenocarcinoma, brain metastases, cerebrospinal fluid, EGFR mutation, droplet digital PCR

## Abstract

**Background:**

We aimed to investigate the feasibility of detecting epidermal growth factor receptor (EGFR) mutations in cell-free DNA (cfDNA) from cerebrospinal fluid (CSF) and plasma of advanced lung adenocarcinoma (LADC) with brain metastases (BMs) by droplet digital polymerase chain reaction (ddPCR).

**Methods:**

Thirty advanced LADC patients with BMs were enrolled, and their matched CSF and plasma samples were collected. Droplet digital PCR was used to test cfDNA in CSF and plasma for EGFR mutation status. The clinical response and prognosis were evaluated.

**Results:**

Out of 30 patients, there were 21 females and 9 males, aged 34-75 years. In all of the cases, CSF cytology were negative. In ddPCR assays, 10 patients (33.3%) had EGFR mutation in CSF, including 3 cases of EGFR T790M mutation, and 16 patients (53.3%) had EGFR mutation in plasma, including 6 cases of EGFR T790M mutation. Five patients with activating EGFR mutations in CSF achieved an intracranial partial response (iPR) after combination treatment with the first-generation EGFR-tyrosine kinase inhibitors. Three patients with EGFR T790M mutations in CSF achieved iPR after second-line osimertinib treatment. The median overall survival and intracranial progression-free survival were 17.0 months and 11.0 months, respectively.

**Conclusion:**

It was feasible to test EGFR mutation in cerebrospinal fluid and plasma. In LADC patients with brain metastasis, cerebrospinal fluid can be used as a liquid biopsy specimen to guide treatment strategy by monitoring EGFR mutation status.

## Introduction

Brain metastases (BMs) occurred in 25-50% of non-small-cell lung cancer (NSCLC) patients (1, 2), 30-60% of those had epidermal growth factor receptor (EGFR) activating mutation lung adenocarcinoma (LADC) (3, 4). Median overall survival of NSCLC patients with BMs ranged from 3 to 15 months in an unselected population without EGFR-tyrosine kinase inhibitors (EGFR-TKIs) targeted therapy (5, 6), and 18 to 58 months in EGFR-mutant patients with EGFR-TKIs treatment (7, 8). EGFR-TKIs have been established as the standard therapy for EGFR-sensitizing mutant (EGFRm, mainly refer to L858R or 19del) advanced NSCLC. In EGFRm patients, first-line EGFR-TKIs treatment has a good response rate of 50 to 80%. However, patients who respond to EGFR-TKIs eventually develop resistance to these drugs, with a median progression-free survival around 9 to 13 months (9).

There are various mechanisms for the development of resistance to EGFR-TKIs. Approximately 50% of the patients who initially respond well to EGFR-TKIs develop resistance due to the occurrence of secondary mutation T790M, an amino acid substitution at position 790 in EGFR from a threonine to a methionine (10, 11). This is the most common mechanism of acquired resistance to EGFR-TKIs. In China, the third-generation EGFR-TKI, osimertinib, is standard treatment for patients with advanced EGFR T790M-mutated NSCLC who have been pre-treated with early-generation EGFR-TKIs (gefitinib, erlotinib, icotinib, or afatinib) (12).

Intracranial progress is the main cause of EGFR-TKIs treatment failure (4, 13). In clinical practice, biopsy of BMs lesions is rarely performed, which results in poor understanding of the resistance mechanisms of EGFR-TKIs therapy in NSCLC with BMs. Liquid biopsy of CSF cfDNA may provide potential information about intracranial lesions. Recent studies have demonstrated that driver and resistance mutations can be identified by droplet digital polymerase chain reaction (ddPCR) or next-generation sequencing (NGS) in CSF circulating cell-free DNA (cfDNA) in patients with central nervous system (CNS) metastases (14–16). Herein, to explore the alternative detection of EGFR mutation status, ddPCR was used to examine the mutation status in CSF and plasma. And the clinical efficacy of EGFR-mutant LADC with BMs was studied based on real clinical practice, including treatment with EGFR-TKIs alone or combined with chemotherapy and radiotherapy.

## Materials and Methods

### Patient Population

Between July 2014 and June 2017 in Beijing Chest Hospital, Capital Medical University (Beijing, China), 30 pathologically confirmed LADC patients with BMs harboring the activating EGFR mutation in their primary tumors were enrolled. The inclusion criteria were as follows: 1) activating EGFR mutation (19del or L858R) in original tissues determined by amplification refractory mutation system polymerase chain reaction (ARMS-PCR); 2) radiological computed tomography or magnetic resonance imaging (MRI) confirmed brain metastases without leptomeningeal metastases; and 3) received lumbar puncture and CSF cytology was negative.

All patients provided written informed consent before specimen collection. This study was reviewed and approved by the institutional review board (IRB)/ethics committee of Beijing Chest Hospital, Capital Medical University.

### Specimen Collection and Processing

CSF samples were obtained by lumbar puncture. Peripheral blood samples were obtained from venous blood. Tumor tissue samples were collected from primary and/or metastatic sites *via* surgical resection or biopsy. The CSF and matched peripheral blood were collected into ethylene diamine tetraacetic acid (EDTA) anti-coagulated tubes from all included subjects. Within 2 h of CSF or peripheral blood sample collection, the sample was placed on ice and centrifuged at 1,000×g at 4°C for 10 min. The CSF supernatant or plasma was transferred to sterilized prelabeled cryotubes, the tubes were stored at -80°C for further exploration.

### Extraction and Quantification of Cell-Free DNA

The 2 mL of CSF or plasma was used for the extraction of cell-free DNA. Stored samples were thawed at room temperature and then centrifuged at 10,000×g at 4 °C for 30 min to remove residual precipitated cellular components and various particles. Circulating cell-free DNA was extracted according to the procedure of the QIAamp Circulating Nucleic Acid Kit (QIAGEN, Hilden, Germany). The concentration of cfDNA was measured with the Qubit dsDNA HS Assay kit (Invitrogen, Life Technologies, CA, USA) on a Qubit 3.0 Fluorometer (Invitrogen, Life Technologies, CA, USA) following manufacturer’s instructions.

### EGFR Mutation Analysis

#### ARMS-PCR for Tissue EGFR Mutations

The initial tissue EGFR mutations were detected by ARMS-PCR with the AmoyDx Human EGFR Gene Mutation Fluorescence PCR Diagnostic Kit (Amoy Diagnostics, Xiamen, China), which had been approved by the National Medical Products Administration for *in vitro* diagnostics use. This kit can cover the 29 most common types of EGFR mutations in exons 18 to 21 of lung cancers, including T790M, L858R, L861Q, S768I, and G719X point mutation; three insertions in exon 20; and 19 deletions in exon 19 (19del). All the experiments were carried out according to the manufacturer’s protocols.

#### Droplet Digital PCR for cfDNA EGFR Mutations

We only detected EGFR 19del, L858R, and T790M mutations for each specimen by ddPCR, and the experiments were carried out at Amoy Diagnostics Co., Ltd (Xiamen, China). Droplet digital PCR was performed using the QX200 AutoDG Droplet Digital PCR System (BioRad, Hercules, CA, USA) according to the manufacturer’s protocol. The method of ddPCR assays has been reported previously and the established sensitivity was 0.04% (17). In short, the ddPCR detection platform can produce about 20,000 droplets of mutant and wild-type DNA emulsion, and the PCR reaction can be carried out in individual droplets. After PCR reaction, positive or negative fluorescence signals were produced in each droplet, indicating whether an EGFR mutant existed or not. In EGFR 19del detection, a 15-base pair peptide nucleic acid (PNA) was introduced to block the amplification of wild-type alleles by targeting the common 19del region, E746 to A750. The FAM-labeled probes were targeted at wild-type and mutant allele amplicons of EGFR exon 19 to reflect deletion mutants in the PNA targeting region. A VIC-labeled probe was designed to target EGFR exon 2 for total EGFR gene input control. The 19 common types of EGFR 19del in the ARMS-PCR kit were all detected by ddPCR analysis. EGFR L858R and T790M were detected by a FAM-labeled probe targeting the mutant region and a VIC-labeled probe targeting the wild-type region, respectively. Human genomic DNA was used as negative control to determine the cutoff of allele calling. We used the QuantaSoft software (version 1.6.6.0320; BioRad, Hercules, CA USA) for ddPCR data analysis of the allele calls. In the test of non-template control reaction, random events occurred occasionally in a single droplet. Therefore, samples of at least two droplets in the FAM signal positive region were regarded as mutation positive. Mutations values were reported as mutant allele frequency (MAF), defined as the proportion of mutant to wild-type PCR products in the ddPCR readout.

### Statistical Analysis

All statistical analyses were performed with SPSS version 24.0 (SPSS Inc., Chicago, IL, USA), or GraphPad Prism version 7.0 (GraphPad Software Inc., San Diego, CA, USA). Frequency tabulation and summary statistics provided the characteristics of data distribution. The intracranial objective responses were evaluated for all patients based on the Response Evaluation Criteria in Solid Tumors (RECIST) version 1.1 (18), and the therapeutic response was evaluated as complete response (CR), partial response (PR), stable disease (SD), or progression disease (PD). Fisher**’**s exact method was used to compare intracranial objective responses (CR+PR versus SD+PD) between EGFR status in different kinds of liquid samples. Kaplan-Meier estimation was used to designate progress-free survival (PFS) and overall survival (OS), and the significant difference was determined by the log-rank test. OS was calculated from the day of diagnosis of brain metastasis to the day of death. Intracranial PFS was calculated from the date of diagnosis of brain metastasis until the date of progression of previous lesions or the appearance of a new lesion. A two-sided *p* value less than 0.05 was considered statistically significant.

## Results

### Patient Characteristics

All of the 30 included patients were Chinese with histologically confirmed lung adenocarcinoma, and brain metastases (BMs) were diagnosed by imaging. At the time of diagnosis with BMs, in all of cases, CSF cytology were negative and examinations of cranial imaging showed no leptomeningeal metastases (LMs). The median age was 58 years (range, 34 to 75 years); nine patients were male and 21 patients were female. The majority (n = 24; 80.0%) were given a good Eastern Cooperative Oncology Group performance status (ECOG PS < 2). Most patients had four or more brain lesions (n = 23; 76.7%). At the time of initial diagnosis with BMs, 19 patients had received no prior treatment, 10 had received first-generation EGFR-TKIs (gefitinib, erlotinib, or icotinib), and one patient had received chemotherapy alone. After diagnosis with BMs, all the patients received systemic treatments, including 20 patients with EGFR-TKIs alone (five cases with second-line EGFR-TKI of osimertinib), six patients with chemotherapy followed by EGFR-TKIs, and four patients with chemotherapy alone. Twenty-one patients had whole brain radiotherapy (WBRT) for local treatment of BMs, one of them also underwent stereotactic radiosurgery (SRS). The baseline clinical characteristics including age, gender, smoking status, ECOG PS, BMs status, and systemic and local treatments are summarized in **Table 1** and detailed case by case in **Supplementary Table 1**.

**Table 1:** Clinicopathologic characteristics of 30 patients.

Characteristic	Value or no. of patients	%
Patients	30	
Age, years		
Median	58	
Range	34-75	
Sex		
Male	9	30.0
Female	21	70.0
ECOG PS		
0	3	10.0
1	21	70.0
2	3	10.0
3	3	10.0
Smoking status		
Never	23	76.7
Current	7	23.3
Tumor histology		
Adenocarcinoma	30	100.0
Primary tissue EGFR status		
19del	18	60.0
L858R	12	40.0
No. of brain metastases		
≤3	7	23.3
>3	23	76.7
BMs at the time of diagnosis		
Yes	19	63.3
No	11	36.7
First-generation TKI treatment		
Prior BMs	10	33.3
Post BMs	20	66.7
BMs local treatment		
WBRT ± SRS	21	70.0
None	9	30.0

No., number; ECOG PS, Eastern Cooperative Oncology, Group performance score; EGFR, epidermal growth factor receptor; BMs, brain metastasis; TKI, tyrosine kinase inhibitor; WBRT, whole brain radiotherapy; SRS, stereotactic radiosurgery.

### EGFR Mutation Status in Tumor Tissue and Liquid Samples

EGFR mutations were detected in primary tumor tissues by ARMS-PCR assays, tissue EGFR 19del mutations were identified in 18 cases (60.0%) and EGFR L858R mutations were detected in 12 patients (40.0%).

Droplet digital PCR assays were performed for paired liquid samples, CSF and plasma samples. In the CSF samples, EGFR mutations were present in 10 patients (33.3%), the more common mutation was 19del (six patients), followed by L858R mutation (four patients). To our surprise, there were three cases (patients 15, 17, and 29) with EGFR T790M mutation in CSF (two accompanied with 19del, one accompanied with L858R). In plasma samples, EGFR mutations were identified in 16 patients (53.3%), including six cases with EGFR T790M mutation (three patients with L858R mutation, two patients with 19del mutation, and one patient with T790M mutation alone). In total, 19 patients found EGFR mutation in CSF or in plasma, no EGFR mutation was found in cerebrospinal fluid or plasma in 11 patients (**Table 2**). All EGFR T790M mutations (nine samples in seven patients) were found during or after EGFR-TKIs treatments.

**Table 2:** EGFR testing result.

Patient	Initial primary tissue EGFR mutation*	CSF EGFR mutation		Plasma EGFR mutation	
		Status	MAF	Status	MAF
**Liquid biopsy at the time of diagnosis with brain metastasis**					
1	19del	WT		T790M	0.3%
2	19del	WT		19del	5.3%
3	L858R	L858R	37.9%	L858R	2.0%
4	L858R	WT		WT	
5	19del	WT		WT	
7	L858R	WT		WT	
8	19del	19del	69.7%	19del/T790M	11.0%/7.0%
11	19del	WT		WT	
13	L858R	L858R	32.8%	WT	
15	19del	19del/T790M	13.2%/0.5%	19del/T790M	14.4%/3.5%
16	L858R	WT		WT	
18	L858R	WT		WT	
19	19del	19del	43.3%	19del	14.9%
21	19del	WT		19del	3.7%
22	19del	19del	21.8%	19del	7.4%
23	L858R	WT		L858R	20.5%
24	19del	WT		19del	11.4%
25	19del	WT		WT	
26	19del	WT		19del	0.8%
27	L858R	L858R	7.2%	L858R	5.2%
28	19del	19del	6.9%	WT	
29	19del	19del/T790M	35.7%/12.1%	WT	
30	19del	WT		19del	15.3%
**Liquid biopsy after brain metastasis progression**					
6	L858R	WT		L858R/T790M	10.8%/26.5%
9	L858R	WT		WT	
10	19del	WT		WT	
12	L858R	WT		L858R/T790M	2.4%/0.2%
14	19del	WT		WT	
17	L858R	L858R/T790M	16.2%/2.0%	L858R/T790M	8.1%/2.2%
20	19del	WT		WT	

CSF, cerebrospinal fluid; EGFR, epidermal growth factor receptor; MAF, mutant allele frequency; WT wild-type.

*EGFR mutations were detected in primary tumor tissues at the time of initial diagnosis with lung cancer by amplification refractory mutation system polymerase chain reaction (ARMS-PCR) assays.

### Correlation Between EGFR Mutation in Liquid Samples and Clinical Responses of BMs

In 30 patients, the best intracranial response rates were 3.3% CR (n = 1), 60.0% PR (n = 18), 30.0% stable disease (n = 9), and 6.7% PD (n = 2).

**Figure 1:**
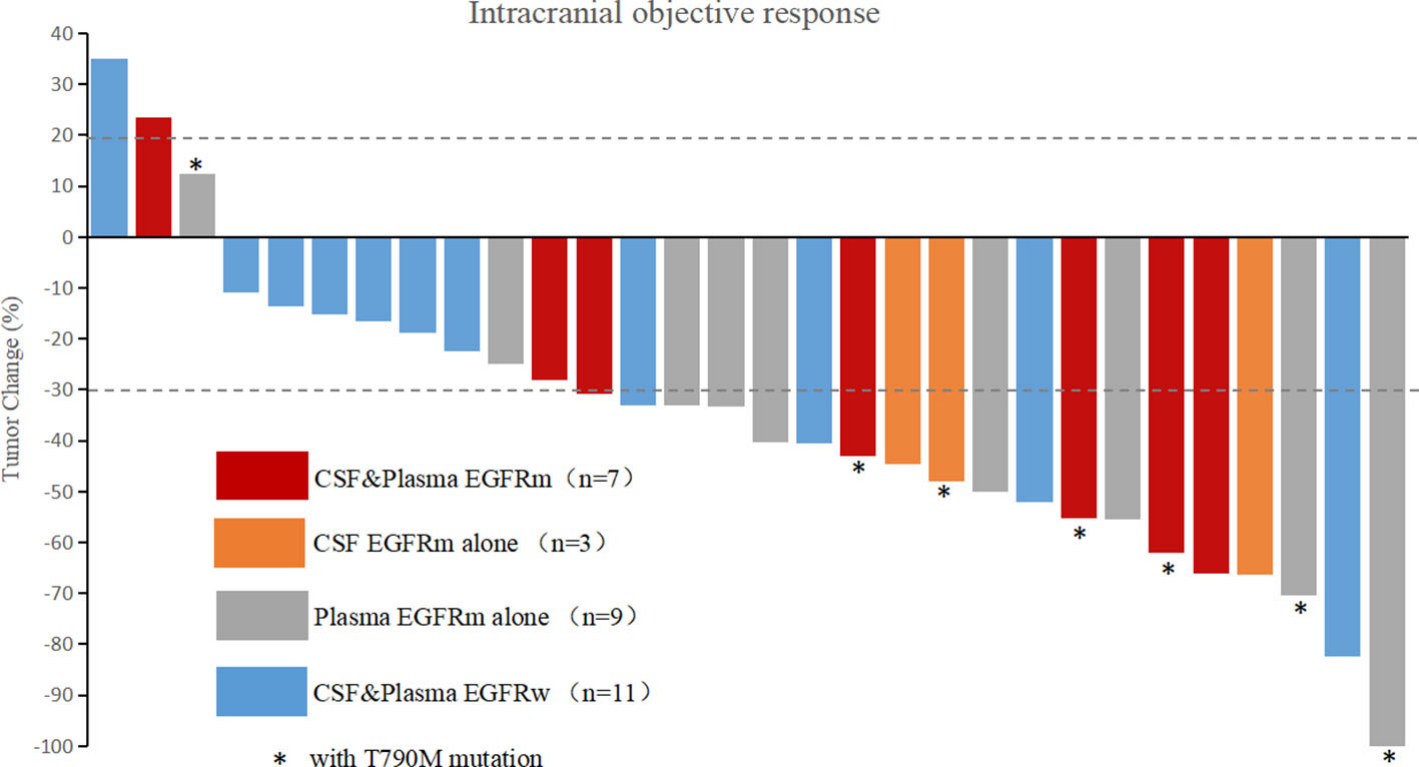
Waterfall plot of intracranial best response. CSF, cerebrospinal fluid; EGFRm, mutant epidermal growth factor receptor; EGFRw, wild-type epidermal growth factor receptor; T790M, an amino acid substitution at position 790 in EGFR from a threonine to a methionine.

EGFR mutations were found in the CSF samples of 10 patients, the five patients with activating EGFR mutations (19del or L858R) achieved intracranial partial response (iPR) after treatment with a combination of WBRT and first-generation EGFR-TKIs, and three patients (patient 15, 17, and 29) with the EGFR T790M mutation were identified after first-generation EGFR-TKIs treatments, then achieved iPR after treatment with second-line osimertinib alone (**Figure 1**). One patient (patient 22) with an EGFR 19del mutation in CSF received first-line gefitinib, and intracranial lesions were stable, the other patient (patient 3) with an EGFR L858R mutation in CSF received two lines of chemotherapy before BMs, and was then treated with gefitinib, but the intracranial lesion progressed.

In liquid samples (CSF or plasma), the RECIST rates of CR, PR, SD, and PD were 5.3%, 73.7%, 15.8%, and 5.3% for patients with EGFRm (in CSF or plasma) and 0%, 36.4%, 54.5%, and 9.1% for patients with wild-type EGFR (EGFRw, in CSF and plasma). The best intracranial response rate (CR+PR) was 78.9% for patients with EGFRm versus 36.4% for patients with EGFRw. There was a significant difference when the numbers of the two groups were compared (CR+PR versus SD+PD, *p* = 0.047).

The waterfall plot of intracranial objective response is shown in **Figure 1**, and the RECIST rates of CR, PR, SD, and PD according to EGFR mutation status of CSF and plasma samples are listed in **Table 3** and detailed in **Supplementary Table 2**.

**Table 3:** Summary of intracranial objective response in different EGFR mutation status.

Intracranial Response	n (%)	CSF EGFR		P*	Plasma EGFR		P*	CSF/plasma EGFR		P*
		Mut, n (%)	Wt, n (%)		Mut, n (%)	Wt, n (%)		Mut, n (%)	Wt, n (%)	
ALL	30 (100.0)	10 (33.3)	20 (66.7)		16 (53.3)	14 (46.7)		19 (63.3)	11 (36.7)	
CR+PR	19 (63.3)	8 (80.0)	11 (55.0)	0.247	12 (75.0)	7 (50.0)	0.257	15 (78.9)	4 (36.4)	0.047
CR	1 (3.3)	0 (0)	1 (5.0)		1 (6.3)	0 (0)		1 (5.3)	0 (0)	
PR	18 (60.0)	8 (80.0)	10 (50.0)		11 (68.8)	7 (50.0)		14 (73.7)	4 (36.4)	
SD+PD	11 (36.7)	2 (20.0)	9 (45.0)		4 (25.0)	7 (50.0)		4 (21.1)	7 (63.6)	
SD	9 (30.0)	1 (10.0)	8 (40.0)		3 (18.8)	6 (42.9)		3 (15.8)	6 (54.5)	
PD	2 (6.7)	1 (10.0)	1 (5.0)		1(6.3)	1 (7.1)		1 (5.3)	1 (9.1)	

EGFR, epidermal growth factor receptor; CSF, cerebrospinal fluid; Mut, mutant; Wt, wild-type; CR, complete response; PR, partial response; SD, stable disease; PD, progression disease.

*P value was assessed by using Fisher’s exact test to compare the number of two groups (CR+PR versus SD+PD).

### Correlation Between EGFR Mutation in Liquid Samples and Prognosis of BMs

**Figure 2:**
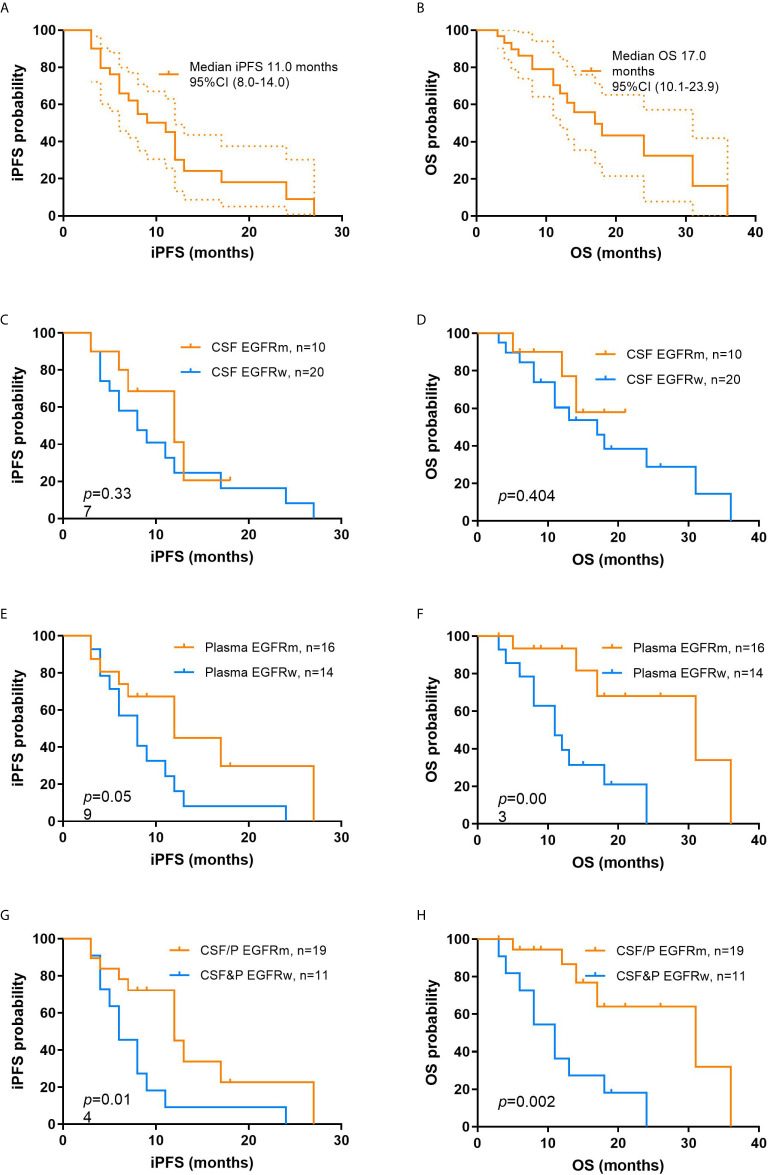
Kaplan-Meier curves of iPFS and OS from the time of diagnosis of brain metastasis in different groups of study. **(A)** iPFS for the overall population; **(B)** OS for the overall population; iPFS **(C)** and OS **(D)** between patients with EGFRm and EGFRw in CSF; iPFS **(E)** and OS **(F)** between patients with EGFRm and EGFRw in plasma; iPFS **(G)** and OS **(H)** between patients with EGFRm and EGFRw in CSF and plasma. iPFS, intracranial progression-free survival; OS, overall survival; EGFRm, mutant epidermal growth factor receptor; EGFRw, wild-type epidermal growth factor receptor; CSF, cerebrospinal fluid.

Fourteen patients were alive at the time of this analysis. The median iPFS and OS from the time of diagnosis with BMs were 11.0 months and 17.0 months, respectively (**Figures 2A, B**).

To emphasize the clinical significance of different EGFR mutation status in liquid samples (EGFRm vs EGFRw), prognosis survival was also evaluated. In CSF samples, the median iPFS of EGFRm and EGFRw were 12.0 months and 8.0 months, respectively (*p* = 0.337), and the median OS of EGFRm and EGFRw were not reached and 17.0 months, respectively (*p* = 0.404; **Figures 2C, D**). In plasma samples, the median iPFS of EGFRm and EGFRw were 12.0 months and 8.0 months, respectively (*p* = 0.059), and the median OS of EGFRm and EGFRw were 31.0 months and 11.0 months, respectively (*p* = 0.003; **Figures 2E, F**). In liquid samples, the median iPFS of EGFRm (CSF or plasma) and EGFRw (CSF and plasma) were 12.0 months and 6.0 months, respectively (*p* = 0.014), and the median OS of EGFRm (CSF or plasma) and EGFRw (CSF and plasma) were 31.0 months and 11.0 months, respectively (*p* = 0.002; **Figures 2G, H**).

### A Case Presentation

**Figure 3:**
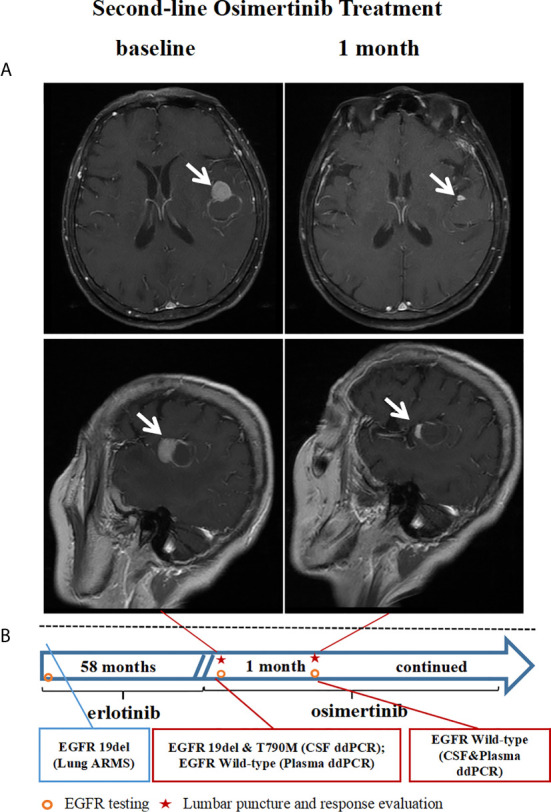
Case presentation: A case received second-line osimertinib treatment after a CSF EGFR T790M mutation was identified by ddPCR. **(A)** Brain MRI imaging tests before and after osimertinib treatment. **(B)** The timeline and results of EGFR mutation status identified by ddPCR. CSF, cerebrospinal fluid; EGFR, epidermal growth factor receptor; T790M, an amino acid substitution at position 790 in EGFR from a threonine to a methionine; ddPCR, droplet digital PCR; MRI, magnetic resonance imaging.

A 66-year-old woman (patient 29) was diagnosed with stage Ia lung adenocarcinoma at disease baseline and underwent a radical right upper lobectomy with video-assisted thoracoscopic surgery (VATS) in February 2010, EGFR 19del was discovered in the primary lung lesion by ARMS-PCR. In January 2011, the tumor recurred and one of the left ribs was involved. After regional radiotherapy of the involved rib, she responded to erlotinib for 58 months before developing brain metastasis (**Figure 3A**) with a central nervous system (CNS) symptom of intermittent headaches. A brain MRI did not shown any evidence of LMs and her cerebrospinal fluid pressure was in the normal range. Tumor cells were not identified in CSF. EGFR 19del and T790M mutations were identified by ddPCR in the CSF sample (**Table 2**, **Figure 3A**), while the mutations were not found in a blood sample at the same time. The patient received second-line osimertinib and achieved intracranial PR after one month of treatment (**Figure 3**). At this time point, the EGFR mutations were not found either in the CSF or in plasma sample.

## Discussion

It is feasible to detect EGFR mutations of CSF cfDNA by ddPCR in advanced NSCLC patients with brain metastasis. As one of the PCR methods, ddPCR is more sensitive than NGS methods and ARMS-PCR for a low abundance of DNA (19). Our results show that the ddPCR method may be suitable for detecting low abundance mutation DNA in CSF. In our study, with the use of ddPCR, we found that CSF EGFR mutations were identified in one third of 30 included cases, and EGFR T790M mutations were found in three patients. To emphasize an important point, all the patients in this study had been confirmed to have brain metastases without leptomeningeal metastases by radiological magnetic resonance imaging or computed tomography, and CSF cytology of all cases were negative. Previous studies on the detection of EGFR mutations in cerebrospinal fluid mainly included patients with leptomeningeal metastases. In 2016, Zhao et al. studied seven patients with leptomeningeal metastases to test EGFR mutations in paired CSF and plasma samples (20). In a recent study, regardless of CSF cytology results, EGFR mutations were detected by NGS in 100% of CSF cfDNA in 26 cases with leptomeningeal metastases of EGFR-mutant NSCLC. However, high-confidence somatic alterations by NGS were found in all 16 (100%) patients with positive CSF cytology and 4 of the 16 (25%) with negative CSF cytology with radiographic evidence for CNS metastases (21). In a similar study on EGFR status in patients with neoplastic meningitis by DNA sequencing, EGFR mutations were reported in 45% of the patients with positive CSF cytology and 30% of the patients with negative CSF cytology (22). Our findings indicate that analysis of CSF cfDNA can be useful for monitoring relevant molecular pathological information of patients with negative tumor cytology in CSF.

Due to the existence of the blood-brain barrier, CSF cfDNA can not be fully circulated in the blood system, thus, plasma can not fully represent the ‘real world’ of intracranial lesions (23). In 10 EGFR mutant cases of CSF, 70% of samples had a concordant EGFR status in their paired plasma samples. Importantly, in one case, T790M mutations were identified in a CSF sample, while the mutations were not found in a blood sample at the same time. We were unable to compare the EGFR status of cerebrospinal fluid samples and intracranial tumors due to the absence of intracranial metastatic tumor tissue. In our study, EGFR mutation detection rate was lower in CSF (33.3%) than that in plasma (53.3%). This is partly due to lower levels of cfDNA in cerebrospinal fluid than in blood. Though recent reports have demonstrated that circulating tumor DNA (ctDNA) was more abundant in CSF than that in plasma of breast cancer (24). In 2 ml of the liquid samples of this study, lower overall cfDNA yields were obtained from CSF (mean ± SD, plasma: 64.59 ± 41.25 ng versus CSF: 23.70 ± 9.52 ng. **Supplementary Figure 1**).

Initial detection of EGFR mutations is necessary to guide TKI treatment, and EGFR mutation status of cfDNA in plasma correlates to TKI response, PFS, as well as OS (25, 26). In this study, 71.4% (5/7) of patients with activating EGFRm (19del or L858R) in CSF samples achieved iPR after treatment with a combination of WBRT and first-generation EGFR-TKIs, and 100% (3/3) of patients with an EGFR T790M mutation achieved iPR after treatment with second-line osimertinib alone. The median iPFS and OS of EGFRm were 12.0 months and 8.0 months, respectively, which were numerically superior to that of EGFRw, however no statistical difference was reached. When we combined the EGFR mutation results of CSF and that of plasma for analysis, the best intracranial response rate (CR+PR) was 78.9% for patients with EGFRm (CSF or plasma) versus 36.4% for patients with EGFRw (CSF and plasma). There was a significant difference. The median iPFS of EGFRm (CSF or plasma) and EGFRw (CSF and plasma) were 12.0 months and 6.0 months, respectively (*p* = 0.014), and the median OS of EGFRm (CSF or plasma) and EGFRw (CSF and plasma) were 31.0 months and 11.0 months, respectively (*p* = 0.002). The accurate identification of tumors with sensitized EGFR mutations, the most common targetable molecular alteration in lung adenocarcinoma, and acquired drug resistance mutations during treatment is a clinical priority. In a recent study, Huang et al. enrolled 35 patients with central nervous system metastases, (including 20 brain metastases and 15 leptomeningeal metastases) to investigate EGFR mutational status in cfDNA from paired CSF and plasma samples. In brain metastases patients, sensitizing EGFR mutations in the CSF or plasma were detected in 5/10 (50%) and 6/11 (54.5%) cases, and EGFR T790M mutations in the CSF or plasma were found in 0/10 (0%) and 4/11 (36.4%) cases (27). The EGFR T790M mutation is the most common mechanism of acquired resistance to first- and second-generation EGFR-TKIs, being present in 50%-60% of the cases (10, 11, 28). The EGFR T790M mutation can be detected accurately by liquid biopsy, and the presence of any detectable T790M ctDNA may be clinically relevant (29, 30). T790M status by liquid biopsy is well correlated with the response of third-generation TKIs (12, 31, 32). Dynamic repeat testing may provide more information about the mechanism of resistance. In this study, we found three cases with a T790M mutation from CSF (including one with wild-type EGFR in a paired blood sample), and all three patients achieved iPR after treatment with second-line osimertinib alone.

There were some limitations to this study. Firstly, this study was a single center retrospective study with a relatively small sample size, resulting in a low statistical power to detect associations. Secondly, as BM lesions biopsies were invasive and difficult to access, we were unable to compare the EGFR genetic profiles between intracranial tissue and CSF. Thirdly, cerebrospinal fluid sampling was 2 ml, and cfDNA yields were relatively few, which may affect the EGFR information of cfDNA. Finally, given the limitations of ddPCR, we only studied the T790M mutation in CSF, which was the most common drug resistance mechanism of first- and second-generation EGFR-TKIs, the other resistance mechanisms were not detected. NGS of cfDNA from CSF may be a better choice for comprehensive genetic profiles to explore the mechanisms of resistance beyond the T790M mutation.

In conclusion, our study demonstrates that it is feasible to test EGFR mutation in CSF and plasma. In LADC patients with brain metastasis, cerebrospinal fluid can be used as a liquid biopsy specimen to guide the treatment strategy by monitoring EGFR mutation status. For advanced LADC patients with BMs harboring EGFR mutation, dynamically monitoring the EGFR mutation status of CSF will be an appropriate choice.

## Data Availability Statement

The original contributions presented in the study are included in the article/**Supplementary Material**. Further inquiries can be directed to the corresponding author.

## Ethics Statement

The studies involving human participants were reviewed and approved by Beijing Chest Hospital, Capital Medical University. The patients/participants provided their written informed consent to participate in this study. Written informed consent was obtained from the individual(s) for the publication of any potentially identifiable images or data included in this article.

## Author Contributions

ZL, LS, and NC conceived and designed the research. LS, JT, HT, LG, WHW, HW, ZCL, LT, WW, HL, QM, LX, and ZL performed the measurements or analyzed the results. LS and ZL wrote the paper. All authors contributed to the article and approved the submitted version.

## Funding

This work was supported by Beijing Municipal Administration of Hospitals Youth Talent Training Project [Grant number: QML20151502] and Scientific Research Common Program of Beijing Municipal Commission of Education [Grant number: KM201610025024].

## Conflict of Interest

The authors declare that the research was conducted in the absence of any commercial or financial relationships that could be construed as a potential conflict of interest.
