# Supplementary material for: Detection of EGFR Mutations in Cerebrospinal Fluid of EGFR-Mutant Lung Adenocarcinoma With Brain Metastases
Source: Front Oncol. 2021 Mar 22;11:622142. doi: 10.3389/fonc.2021.622142 (PMC8019917; doi:10.3389/fonc.2021.622142)
Supplement: Supplementary file 1 [file DataSheet_1.docx]

Supplementary Figure 1: Overall cell-free DNA (cfDNA) yields were obtained from 2 mL cerebrospinal fluid (CSF) and plasma.


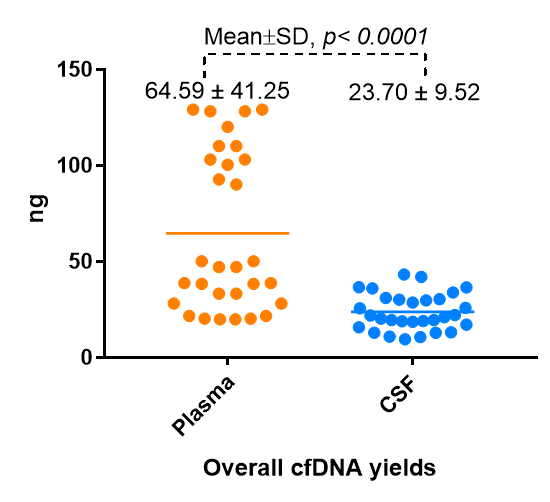


Supplementary Table 1. Characteristics of 30 Patients with BMs

| **Patient** | **Gender** | **Age (years)** | **Smoking  Status** | **ECOG PS at BMs** | **Initial Diagnosis with BMs** | **No. of BMs** | **Systemic Treatment before BMs** | **Systemic Treatment after BMs** | **BMs Radiotherapy** |
| --- | --- | --- | --- | --- | --- | --- | --- | --- | --- |
|  |  |  |  |  |  |  |  |  |  |
| 1 | Female | 34 | Never | 1 | No | ≤3 | Erlotinib→CT | CT | WBRT+SRS |
| 2 | Female | 60 | Never | 0 | No | >3 | CT→Erlotinib | CT | WBRT |
| 3 | Female | 54 | Current | 1 | No | >3 | CT | Gefitinib | WBRT |
| 4 | Female | 56 | Never | 1 | No | >3 | CT→Gefitinib | CT | WBRT |
| 5 | Female | 48 | Never | 1 | No | ≤3 | CT→Gefitinib | CT | WBRT |
| 6 | Female | 44 | Never | 1 | Yes | >3 | NA | CT→Erlotinib | WBRT |
| 7 | Female | 60 | Never | 1 | No | >3 | Gefitinib | Gefitinib | WBRT |
| 8 | Female | 36 | Never | 1 | No | ≤3 | Gefitinib | Osimertinib | WBRT |
| 9 | Male | 56 | Never | 2 | Yes | ≤3 | NA | CT→Erlotinib | None |
| 10 | Male | 49 | Never | 1 | Yes | >3 | NA | CT→Erlotinib | WBRT |
| 11 | Female | 60 | Never | 1 | Yes | >3 | NA | Icotinib | WBRT |
| 12 | Male | 50 | Current | 1 | Yes | >3 | NA | CT→Icotinib | WBRT |
| 13 | Female | 74 | Never | 3 | Yes | >3 | NA | Icotinib | WBRT |
| 14 | Female | 65 | Never | 3 | Yes | >3 | NA | CT→Gefitinib | WBRT |
| 15 | Female | 63 | Never | 1 | No | ≤3 | CT→Gefitinib | Osimertinib | None |
| 16 | Female | 72 | Never | 2 | No | >3 | Icotinib | Icotinib | WBRT |
| 17 | Male | 63 | Current | 1 | Yes | >3 | NA | Gefitinib→Osimertinib | None |
| 18 | Female | 75 | Never | 1 | Yes | >3 | NA | Gefitinib | None |
| 19 | Female | 71 | Never | 1 | Yes | >3 | NA | Icotinib | WBRT |
| 20 | Female | 65 | Current | 1 | Yes | >3 | NA | CT→Gefitinib | WBRT |
| 21 | Female | 62 | Never | 0 | Yes | >3 | NA | Gefitinib | WBRT |
| 22 | Male | 34 | Current | 1 | Yes | >3 | NA | Gefitinib | WBRT |
| 23 | Male | 51 | Never | 1 | Yes | >3 | NA | Gefitinib | WBRT |
| 24 | Female | 65 | Never | 2 | Yes | >3 | NA | Erlotinib | None |
| 25 | Male | 71 | Current | 3 | Yes | >3 | NA | Gefitinib | None |
| 26 | Male | 52 | Current | 0 | Yes | >3 | NA | Gefitinib | WBRT |
| 27 | Female | 54 | Never | 1 | Yes | ≤3 | NA | Gefitinib | WBRT |
| 28 | Male | 40 | Never | 1 | No | >3 | Gefitinib | Osimertinib | None |
| 29 | Female | 66 | Never | 1 | No | ≤3 | Erlotinib | Osimertinib | None |
| 30 | Female | 43 | Never | 1 | Yes | >3 | NA | Icotinib | None |

Abbreviations: No., number; ECOG PS, Eastern Cooperative Oncology Group performance score; EGFR-TKI, epidermal growth factor receptor tyrosine kinase inhibitor; BMs, brain metastasis; CT, chemotherapy; WBRT, whole brain radiotherapy; SRS, Stereotactic radiosurgery; NA, Not applicable.

Supplementary Table 2. Detailed Intracranial Objective Response and Treatment Information in CSF and Plasma EGFR Mutation Status

| **Patient** | **CSF EGFR Status** | **Plasma EGFR Status** | **Systemic Treatment after BMs** | **BMs Radiotherapy** | **Intracranial Response** |
| --- | --- | --- | --- | --- | --- |
|  |  |  |  |  |  |
| 1 | WT | T790M | CT | WBRT+SRS | PR |
| 2 | WT | 19del | CT | WBRT | PR |
| 3 | L858R | L858R | Gefitinib | WBRT | PD |
| 4 | WT | WT | CT | WBRT | SD |
| 5 | WT | WT | CT | WBRT | PR |
| 6 | WT | L858R/T790M | CT→Erotinib | WBRT | CR |
| 7 | WT | WT | Gefitinib | WBRT | PR |
| 8 | 19del | 19del/T790M | Osimertinib | WBRT | PR |
| 9 | WT | WT | CT→Erotinib | None | PR |
| 10 | WT | WT | CT→Erotinib | WBRT | PR |
| 11 | WT | WT | Icotinib | WBRT | SD |
| 12 | WT | L858R/T790M | CT→Icotinib | WBRT | SD |
| 13 | L858R | WT | Icotinib | WBRT | PR |
| 14 | WT | WT | CT→Gefitinib | WBRT | SD |
| 15 | 19del/T790M | 19del/T790M | Osimertinib | None | PR |
| 16 | WT | WT | Icotinib | WBRT | SD |
| 17 | L858R/T790M | L858R/T790M | Gefitinib→Osimertinib | None | PR |
| 18 | WT | WT | Gefitinib | None | SD |
| 19 | 19del | 19del | Icotinib | WBRT | PR |
| 20 | WT | WT | CT→Gefitinib | WBRT | SD |
| 21 | WT | 19del | Gefitinib | WBRT | SD |
| 22 | 19del | 19del | Gefitinib | WBRT | SD |
| 23 | WT | L858R | Gefitinib | WBRT | PR |
| 24 | WT | 19del | Erotinib | None | PR |
| 25 | WT | WT | Gefitinib | None | PD |
| 26 | WT | 19del | Gefitinib | WBRT | PR |
| 27 | L858R | L858R | Gefitinib | WBRT | PR |
| 28 | 19del | WT | Osimertinib | None | PR |
| 29 | 19del/T790M | WT | Osimertinib | None | PR |
| 30 | WT | 19del | Icotinib | None | PR |

Abbreviations: CSF,cerebrospinal fluid; EGFR, epidermal growth factor receptor; WT, wild-type; BMs, brain metastasis; CT, chemotherapy; WBRT, whole brain radiotherapy; SRS, Stereotactic radiosurgery; CR, complete response; PR,partial response; SD, stable disease; PD, progression disease.
